# A *Kayvirus* Distant Homolog of Staphylococcal Virulence Determinants and VISA Biomarker Is a Phage Lytic Enzyme

**DOI:** 10.3390/v12030292

**Published:** 2020-03-07

**Authors:** Aleksandra Głowacka-Rutkowska, Magdalena Ulatowska, Joanna Empel, Magdalena Kowalczyk, Jakub Boreczek, Małgorzata Łobocka

**Affiliations:** 1 Institute of Biochemistry and Biophysics of the Polish Academy of Sciences, 02-106 Warsaw, Poland; glowacka@ibb.waw.pl (A.G.-R.); miulatowska@gmail.com (M.U.); mk@ibb.waw.pl (M.K.); jakub.boreczek@ibb.waw.pl (J.B.); 2 Department of Epidemiology and Clinical Microbiology, National Medicines Institute, 00-725 Warsaw, Poland; j.empel@nil.gov.pl

**Keywords:** *Staphylococcus aureus*, bacteriophage, *Kayvirus*, endolysin, virulence determinants, vancomycin

## Abstract

Staphylococcal bacteriophages of the *Kayvirus* genus are candidates for therapeutic applications. One of their proteins, Tgl, is slightly similar to two staphylococcal virulence factors, secreted autolysins of lytic transglycosylase motifs IsaA and SceD. We show that Tgl is a lytic enzyme secreted by the bacterial transport system and localizes to cell peripheries like IsaA and SceD. It causes lysis of *E. coli* cells expressing the cloned *tgl* gene, but could be overproduced when depleted of signal peptide. *S. aureus* cells producing Tgl lysed in the presence of nisin, which mimics the action of phage holin. In vitro, Tgl protein was able to destroy *S. aureus* cell walls. The production of Tgl decreased *S. aureus* tolerance to vancomycin, unlike the production of SceD, which is associated with decreased sensitivity to vancomycin. In the genomes of kayviruses, the *tgl* gene is located a few genes away from the *lysK* gene, encoding the major endolysin. While *lysK* is a late phage gene, *tgl* can be transcribed by a host RNA polymerase, like phage early genes. Taken together, our data indicate that *tgl* belongs to the kayvirus lytic module and encodes an additional endolysin that can act in concert with LysK in cell lysis.

## 1. Introduction

*Staphylococcus aureus* is one of the most challenging bacterial pathogens because of the increasing number and spread of antibiotic-resistant strains that are a serious threat to health and life [1]. Thus, bacteriophage therapy may become a future option of choice to fight infections with antibiotic-resistant *S. aureus* [2]. As a rule, phages target bacteria of certain strains or species independently of bacterial resistance to antibiotics. Bacteriophages are harmless to eukaryotic cells and propagate in a self-limiting manner, which is controlled by the availability of a sensitive host [3]. Most importantly, bacteriophages specific for certain bacterial pathogens do not destroy natural human or animal microflora and do not cause the selection of antibiotic-resistant strains [4].

A dominant group of staphylococcal phages in therapeutic phage collections is represented by tailed phages of the *Kayvirus* genus of the *Twortvirinae* subfamily, the *Herelleviridae* family [5]. They are obligatorily lytic, and infect a broad spectrum of *S. aureus* strains [6,7]. Several *Kayvirus* genus representatives have been successfully used in experimental antistaphylococcal therapies in humans and animals [2,8,9,10,11,12,13,14]. The genomic sequences of most of them have been determined [6,15]. They vary between 127 kb and 152 kb and do not transfer bacterial DNA by transduction. Core genome regions of kayviruses and other phages of the *Twortvirinae* subfamily are conserved and resemble in organization and coding properties the core genome regions of other phages of *Herelleviridae* family [5,6]. Nearly half of about 200 kayviruses genes have been assigned function or putative function based on homologies or, to a lesser extent, results of experimental studies.

It is commonly accepted that in addition to the obligatorily lytic propagation strategy and the inability to transfer bacterial DNA by transduction, a feature that qualifies phages for potential use in antibacterial therapies is the lack of genes encoding toxins or other virulence determinants, as well as antibiotic resistance markers [16,17]. In our previous work we identified in the genomes of *Kayvirus* genus representatives, a conserved gene (designated by us as *tgl*), whose product is a distant homolog of two staphylococcal virulence determinants, proteins IsaA and SceD [6]. Additional analysis of known or predicted proteins encoded by 22 kayviruses revealed that Tgl is the only gene product of these phages that has slight similarities at the amino acid sequence level to proteins associated with bacterial virulence [17].

IsaA and SceD are paralogous staphylococcal autolysins and surface antigens [18,19]. They are similar in size (231 and 233 amino acid residues, respectively), share 33% of their amino acid sequence, and have a lytic transglycosylase domain motif (pfam 06737) in their C-terminal part. They both contribute to cell wall remodeling, are required for normal growth of *S. aureus*, and are associated with *S. aureus* virulence [18,19,20]. Each of them contributes differently to biofilm formation and beta-lactam resistance, and they have opposite effects on *S. aureus* cell clumping and septation [18,21,22,23,24].

In the genome of *S. aureus*, the *isaA* and *sceD* genes are located in unlinked, monocistronic operons that are far away from each other (see, e.g., [25]) (GB acc. no. NC_007795.1). They both belong to the essential WalKR two-component system regulon (formerly YycFG) controlling cell wall metabolism and are positively regulated by WalR [26,27]. Additionally, they are oppositely controlled by two global regulators of virulence genes, SarA and *agr* [18,28,29,30]. Besides the common factors participating in the control of expression of both genes, *sceD* is positively regulated by sigma B and by two-component regulatory systems LytSR and SaeRS [30]. While inactivation of *isaA* leads to elevated levels of *sceD* expression, the reciprocal effect was not observed, indicating the overlapping and also distinct physiological roles of IsaA and SceD [18,21].

IsaA is a major *S. aureus* immunodominant antigen, which is surface-exposed and has been identified in the exoproteomes of all tested *S. aureus* clinical isolates studied [19,31,32,33,34]. It is bound to *S. aureus* cell wall by non-covalent interactions [35]. Diseases associated with *S. aureus* colonization, including sepsis caused by methicillin-resistant *S. aureus* (MRSA), are associated with increased IgG response against IsaA [36,37,38,39]. Monoclonal anti-IsaA antibodies were effective in the treatment of staphylococcal infections [40,41,42].

SceD is mostly secreted in the growth medium [35]. Its increased production was essential for the establishment of *S. aureus* nasal colonization in rats [18,43]. Moreover, SceD is overproduced in most of the MRSA strains with decreased sensitivity to vancomycin, specifically vancomycin-intermediate *S. aureus* (VISA) and heterogeneous VISA (hVISA). Hence it has been proposed as a potential biomarker for the detection of VISA and hVISA strains [44,45,46,47,48]. Pieper et al. suggested that its action on the cell wall is required to increase tolerance to vancomycin [46].

The similarity of Tgl to IsaA and SceD, albeit limited, poses a question about the role of Tgl protein in the development of kayviruses or their interaction with a bacterial host. In a search for the answer to these questions, we analyze here the extent and significance of Tgl homologies to IsaA and SceD, and show that Tgl is an additional kayvirus endolysin that may possibly contribute to the high lytic efficacy of these phages.

## 2. Materials and Methods

### 2.1. Bacterial Strains and Culture Conditions

The bacterial strains used in this study were *E. coli* DH5α (F^–^ φ80*lac*ZΔM15 Δ(*lac*ZYA-*arg*F) U169 *rec*A1 *end*A1 *hsd*R17(r_K_^–^, m_K_^+^) *pho*A *sup*E44 λ^–^ *thi*-1 *gyr*A96 *rel*A1) [49,50], *E. coli* BL21 (F^–^ *omp*T *hsd*S_B_ (r_B_^–^, m_B_^–^) *gal dcm* (DE3)) [51], and *S. aureus* RN4220, a restriction-deficient derivative of NCTC8325 [52,53]. Bacteria were grown in Luria-Bertani broth (LB; Difco) or in trypticase soy broth (TSB; Difco) with constant shaking (200 rpm) or on agar-solidified LB medium at 30, 37, or 42 °C, where indicated. When required, antibiotics were added to the medium at the following concentrations: 100 µg/mL ampicillin (*E. coli*) or 20 µg/mL chloramphenicol (*S. aureus*). LB solid medium supplemented with 5-bromo-4-chloro-3-indolyl-β-d-galactopyranoside (x-gal; 200 μg/mL) served to detect the production of β-galactosidase by *S. aureus* and *E. coli* cells.

### 2.2. Bacteriophage and Bacteriophage Propagation

Bacteriophage MSA6, a representative member of *Kayvirus* genus phages and a source of *tgl* gene, was described previously [6,54]. MSA6 DNA was isolated from lysates of infected *S. aureus* RN4220 cells following the previously described procedures [6,12].

### 2.3. Plasmids

Shuttle *E. coli*–*S. aureus* expression vectors used in this study are based on a hybrid plasmid, pMLE2, derived from the core sequences of *E. coli*–*S. aureus* shuttle pSK5630 vector, kindly provided by Ron Skurray [55], and the expression cassette of pDAS122 plasmid, kindly provided by David A. Schofield [56] (Figure S1). The cloning site in pMLE2 is preceded by the synthetic promoter (Pro3). Transcription from Pro3 is repressed by the pMLE2-encoded temperature-sensitive C1 repressor of phage P1 (C1-100) and the P1 Bof (Lxc) protein, which enhances the repression [56]. To construct pMLE2, the expression cassette of pDAS122 cut out with StyI and BamHI and blunted at the StyI end was ligated with the SalI- and BamHI-digested pSK5630 blunted at the SalI end. The pMLE2-derived pMLE3 plasmid contains the PmaCI recognition site between the Pro3 promoter and the *lacZ* gene. It was constructed by the religation of PmaCI digested amplicon obtained with pMLE2 DNA and primers OMLS001F and OMLS002R. Plasmid pMLE4 is a derivative of pMLE3 containing the ClaI recognition site in place of *lacZ*. It was constructed by the insertion of hybridized pMLE1F and pMLE2R oligonucleotides in place of the ClaI***-***SphI fragment of pMLE3. In the pMLE4-derived pMLE5 plasmid, the cloning site is enriched with SacI, AgeI, and XbaI recognition regions. It was constructed by the ligation of hybridized pMLE3F and pMLE4R oligonucleotides with the ClaI- and SphI-digested pMLE4. The pMLE5-derived pMLE6 plasmid contains the Egfp*-*encoding gene downstream of the Pro3 promoter. It was constructed by the replacement of AgeI-XbaI fragment of pMLE5 with the AgeI-XbaI fragment of the pEGFP plasmid (Clontech; GenBank acc. no. U76561). The pAGL1 and pAGL2 plasmids are constitutive expression derivatives of pMLE3 and pMLE5, respectively, with the repressor gene (*c1-100*) inactivated by a frameshift mutation. They were constructed by the religation of pMLE3 and pMLE5, respectively, digested with BglII, and blunted at the ends. The pAGL3 plasmid is a pMLE5 derivative containing the *tgl* gene of bacteriophage MSA6 downstream of the Pro3 promoter. It was constructed by the ligation of ClaI- and SphI-digested amplicon obtained with MSA6 DNA and primers OAGL40 and OAGL39, with the ClaI- and SphI-digested pMLE5. The pMLE6-derived pAGL4 plasmid contains the translational fusion of *tgl* gene with the *egfp* gene. It was constructed by the ligation of ClaI- and AgeI-digested amplicon obtained with MSA6 DNA and primers OAGL40 and OAGL49, with the ClaI- and AgeI-digested pMLE6. The pAGL5 plasmid is a derivative of pMLE3 containing the transcriptional fusion of *tgl* gene promoter–operator region with the promotorless *lacZ* gene. It was constructed by the ligation of NarI- and PmaCI-digested amplicon obtained with MSA6 DNA as a template and primers OAGL57 and OAGL78, with the NarI- and PmaCI-digested pMLE3 plasmid. The pAGL6 plasmid is a derivative of pMLE5 carrying the *tgl* gene truncated with 28 codons at its 5’ end and enriched with six histidine codons at its 3' end. It was constructed by the insertion of ClaI- and SphI-digested amplicon obtained with phage MSA6 DNA and primers OAGL108 and OAGL109, in place of ClaI-SphI polylinker fragment of pMLE5. All DNA manipulation procedures were performed according to the standard protocols [57] or as recommended by enzyme suppliers. The sequence correctness of all fragments obtained by PCR was verified by DNA sequencing, with primers OAGL41 and OAGL42. The sequencing was performed at the Laboratory of DNA Sequencing and Oligonucleotides Synthesis of the Institute of Biochemistry and Biophysics, Polish Academy of Sciences. Oligonucleotide sequences used for the DNA amplification or construction of plasmids are listed in Table S1.

### 2.4. Bioinformatics Analysis

Comparative sequence analysis of Tgl protein and gene products of the *tgl-*flanking regions in the genomes of *Kayvirus* genus phages with proteins in databases was performed using Blastp [58], PSI-Blast [59], and Tblastn [58] at the National Center for Biotechnology (NCBI), and HHpred [60]. The N-terminal sequence of the Tgl protein was searched for putative transmembrane domains and signal peptides as described elsewhere [61].

### 2.5. Testing the Influence of Intracellular Tgl Protein on the Growth and Survivability of E. coli and S. aureus Cells

*E. coli* DH5α or *S. aureus* RN4220 cells, bearing the pAGL3 plasmid with the *tgl* gene under the control of thermoinducible Pro3 promoter, were grown overnight in LB supplemented with ampicillin (100 µg/mL, *E. coli*) or chloramphenicol (20 µg/mL, *S. aureus*) at 30 °C. Overnight cultures were diluted 1:100 in similar medium (OD_600_ of about 0.001) and incubated with shaking (200 rpm) at 42 °C to derepress the expression of the cloned *tgl* gene. Bacterial growth was monitored spectrophotometrically (OD_600_). The number of viable cells in each culture was determined based on the number of colony-forming units (CFU/mL) obtained after plating of diluted culture samples and overnight incubation of plates at 30 °C. Cells harboring the empty pMLE5 plasmid were used as control. Each experiment was performed in triplicate.

### 2.6. Determination of the Intracellular Localization of Tgl-Egfp Protein in S. aureus Cells

*S. aureus* RN4220 cells bearing the pAGL6 plasmid, which carries the *tgl*-*egfp* fusion under the control of thermoinducible promoter, were grown overnight in LB supplemented with chloramphenicol at 30 °C. Overnight cultures were diluted 1:100 in similar medium and incubated at 30 °C with shaking (200 rpm) until reaching optical density (OD_600_) of about 0.3. Cultures were then transferred to 42 °C to derepress the expression of cloned *tgl* gene. To visualize the Tgl-Egfp protein in individual cells, bacteria were immobilized on an agarose-padded microscope slide. Briefly, 100 μL of molten (75 °C) agarose solution (1.5% Agarose SeaKem GTG in 0.7% NCl) was dropped on a pre-warmed microscope slide, covered with a cover glass, and left to solidify. Next, the cover glass was removed. Cell culture (1 mL) was harvested by centrifugation (10,000× *g* for 5 min) and resuspended in 100 µL of 1.5% NaCl. Aliquots of cell suspension (10 μL) were dropped on the agarose and coated with a new cover glass. Cells were viewed with an Eclipse TE2000-E confocal microscope equipped with a Nikon DS-5Mc color camera and a CFI Plan Apochromat oil immersion objective (100×, numerical aperture = 1.4) with an additional 10× zoom option. A Nikon module for fluorescence equipped with an argon laser (Melles Griot) and a Nikon BA515-555 filter set was used to excite the fluorescence of Egfp with blue light (488 nm wavelength) and observe the Egfp emission light within the wavelength range of 500–530 nm. Confocal microscopy observations were performed at the Laboratory of Confocal and Fluorescence Microscopy of the Institute of Biochemistry and Biophysics of Polish Academy of Sciences, Warsaw, Poland.

### 2.7. Assays of β-Galactosidase Activity

Bacteria for assays of β-galactosidase activity were grown overnight in LB supplemented with ampicillin (*E. coli*) or chloramphenicol (*S. aureus*) at 30 °C. Overnight cultures were diluted 1:100 in similar medium, incubated at 30 or 42 °C with shaking (200 rpm) until reaching optical density (OD_600_) of about 0.4, and chilled in ice for 15 min to stop growth. *S. aureus* cells for the assays (0.5 mL) were harvested by centrifugation, resuspended in the same volume of lysis buffer (0.01 M potassium phosphate buffer (pH 7.8), 0.015 M EDTA, 1% Triton X-100, 100 mg of lysostaphin per mL), and incubated at 37 °C for 30 min with vigorous shaking. The portions of lysed *S. aureus* cells or *E. coli* cultures were mixed with 0.5 mL of chilled Z buffer (60 mM Na_2_HPO_4_·7H_2_O, 40 mM NaH_2_PO_4_·H_2_O, 10 mM KCl, 1 mM MgSO_4_·7H_2_O, 50 mM β-merkaptoethanol; pH 7.0) to obtain a final volume of 1 mL. Lysis of *E. coli* cells was performed in the reaction mixtures by adding chloroform (50 µL) and 0.1% SDS (25 µL). Next, the probes were vigorously vortexed for 10 s and incubated for 20 min at 28 °C. Reactions were started by adding 0.2 mL of *o*-nitrophenyl-β-d-galactopyranoside (ONPG; freshly prepared solution in H_2_O: 4 mg/mL; Sigma-Aldrich), incubated until the appearance of yellow coloration, and stopped by the addition of 1 M Na_2_CO_3_ (0.5 mL). The enzyme activity was calculated according to Miller’s method [62].

### 2.8. Preparation of S. aureus Cell Walls for Zymography

*S. aureus* cell walls were prepared as described previously [63]. Briefly, overnight culture of *S. aureus* was diluted 1:100 in 250 mL of TSB and incubated at 37 °C with shaking (200 rpm) until reaching optical density (OD_600_) of about 1.0. Next, bacteria were harvested by centrifugation (10,000× *g* for 15 min, room temperature), washed with 250 mL of Milli-Q water, centrifuged as above, and resuspended in 30 mL of Milli-Q water. The cell suspension was autoclaved (15 min, 121 °C) and centrifuged as above, and the pellet obtained was stored overnight at −20 °C. Next, the pellet was resuspended in 3 mL of Milli-Q water and distributed into pre-weighted tubes. The suspension was dried overnight in a speed vac. The dried pellet was weighted, re-suspended in Milli-Q water to reach a final concentration 50 mg/mL, and stored at −20 °C.

### 2.9. Overproduction of TglΔSP Protein and Zymography

Overnight culture of *E. coli* BL21 cells harboring the pAGL6 plasmid, which carries the truncated version of *tgl* gene (*tglΔSP)*, was diluted 1:100 in 100 mL of fresh LB supplemented with ampicillin and incubated at 30 °C with shaking (200 rpm) until reaching optical density (OD_600_) of about 0.4. Cells harboring pMLE5 plasmid (empty vector) were used as controls. Cultures were then transferred to 42 °C to derepress the transcription from the Pro3 promoter. After 5 hours of growth with shaking, the cells were harvested by centrifugation (10,000× *g* for 15 min), resuspended in 1 mL of lysis buffer (50 mM NaH_2_PO_4_, 300 mM NaCl, 10 mM imidazole, 1 mM PMSF, pH 8.0) containing a protease inhibitor cocktail (complete EDTA-free Protease Inhibitor Cocktail; Roche Applied Science), and disrupted by sonication (Bioruptor UCD-200 sonication system, Diagenode, Belgium) with the following operating conditions: power setting: high; sonication cycle: 30 sec ON, 30 sec OFF; total sonication time: 10 min. The cell extracts were centrifuged (10,000× *g* for 30 min, 4 °C) to remove insoluble cell debris. The supernatant obtained was left for further analysis. The pellet was dissolved in 1 mL of lysis buffer with 8 M urea. Next, the 20 µL samples of supernatant or dissolved pellet were mixed with Laemmli buffer (Bio-Rad Laboratories, Inc.), heated at 95 °C for 5 min, and separated electrophoretically in 10% SDS-PAGE gel. Gels were kept in staining solution (InstantBlue, Expedeon) for 1 h at room temperature with gentle shaking, submerged in Milli-Q water, and incubated until the protein bands became visible. For zymography, 20 µL portions of the supernatant or dissolved cell pellet were mixed with Laemmli buffer (Bio-Rad) and loaded (without prior boiling) to 10% SDS-PAGE gel (0.75 mm thickness) containing *S. aureus* cell walls (prepared as above) at a final concentration of 2 mg/mL [63]. Following electrophoresis, the gel was rinsed briefly with Milli-Q water and washed three times with Milli-Q water with gentle agitation for 15 min at room temperature. Then, the gel was incubated overnight at 37 °C with gentle agitation in freshly prepared renaturation buffer (50 mM Tris-HCl (pH 7.5), 0.1% (*v*/*v*) Triton X-100, 10 mM CaCl_2_, 10 mM MgCl_2_). Zymograms were stained in methylene blue solution (0.1% (*w*/*v*) in 0.01% potassium hydroxide) for 1 h and destained in water until the bands formed by transparent gel fragments became clearly visible.

### 2.10. Preparation of Nisin Stock Solution and Nisin Activity Assay

Brain heart infusion (BHI) growth medium (110 mL; Oxoid) supplemented with glucose (0.8% *w*/*v*) was inoculated with 100 µL of fresh o/n culture of the nisin producing *Lactococcus lactis* IBB 1339 strain from the IBB PAS laboratory culture collection. After incubation at 30 °C for 20 h, the culture was centrifuged at 6800× *g* for 15 min to remove cells. Ammonium sulfate (30 g) was added to 100 mL of supernatant and dissolved at room temperature by gentle mixing; the samples were kept in ice for 60 min to allow protein precipitation, then centrifuged at 12,850× *g* for 40 min. The pellet containing nisin was dissolved in 1 mL of sterile Milli-Q water and incubated at 100 °C for 2 min to kill contaminating cells. Nisin concentrate was stored at −20 °C. The activity of nisin in the concentrate was assayed according to the critical dilution method [64] as previously described [65,66]. The *L. lactis* subsp. *lactis* IL1403 strain, which is sensitive to nisin, served as an indicator. Briefly, fresh GM17 medium (Oxoid; composition per 1 L: pancreatic digest of casein 5.0 g, soy peptone 5.0 g, beef extract 5.0 g, yeast extract 2.5 g, ascorbic acid 0.5 g, magnesium sulfate 0.25 g, disodium-β-glycerophosphate 19.0 g) was used to prepare two-fold serial dilutions of the nisin concentrate. Aliquots (10 μL) of each dilution were dropped onto the layer of approximately 10^7^ indicator cells embedded in GM17 soft agar (0.7% *w*/*v*) on the surface of GM17 solid medium in a Petri dish. The nisin activity, expressed in arbitrary units (AU) per mL, was calculated as follows: the highest dilution factor yielding a clear zone of inhibition on the indicator lawn after 18 h incubation was multiplied by 100 to obtain AU/mL. Each assay was performed in triplicate.

### 2.11. Testing the Influence of Tgl Protein Production on the Sensitivity of S. aureus to Vancomycin

The sensitivity of *S. aureus* cells producing Tgl to vancomycin was assayed by determining the minimal inhibitory concentration (MIC) for vancomycin and by monitoring the growth of bacteria in cultures supplemented with vancomycin. The MIC for vancomycin was determined in Muller Hinton broth (MHB; Difco) using the two-fold dilution plate method as described previously [67], with some modifications. Briefly, overnight cultures of *S. aureus* cells bearing pAGL3 (*tgl*+) or pMLE5 plasmid (empty control vector) were diluted 1:100 in MHB supplemented with chloramphenicol (20 µg/mL) and incubated with shaking for 1 h at 30 °C and for additional 1 h at 42 °C (to derepress the expression of cloned *tgl* gene). At zero time, 100 μL portions of cell culture were transferred to wells of prewarmed (42 °C) honeycomb plates containing 100 μL MHB with or without vancomycin. MHB medium (200 µL) without antibiotic served as a control for medium sterility. The plates were incubated for 5 h at 42 °C. After incubation, 66 µL of dimethyl thiazolyl diphenyltetrazolium bromide (MTT) (0.3 mg/mL) or triphenyl tetrazolium chloride (TTC) (0.1%) per well was added and the plates were incubated for 90 min at 37 °C with gentle shaking (110 rpm). The MICs were read manually, based on the changes of natural colors of MTT and TTC diluted in MHB from the colors of their reduced forms.

To monitor the growth of *S. aureus* producing Tgl in the presence of vancomycin, overnight cultures of *S. aureus* RN4220 cells bearing pAGL3 (*tgl*+) or pMLE5 plasmid (empty control vector) were diluted 1:100 in MHB supplemented with chloramphenicol (20 µg/mL) and incubated with shaking for 1 h at 30 °C and for an additional 1 h at 42 °C (to derepress the expression of cloned *tgl* gene). At time zero, 100 μL portions of culture were transferred to wells of pre-warmed (42 °C) honeycomb plates containing 100 μL of MHB supplemented with vancomycin at various concentrations. The plates were incubated in a Bioscreen C Microbiology Plate Reader (Growth Curves USA, Piscataway, NJ, USA) for 6 h at 42 °C with medium-intensity shaking. The optical density of samples (OD_600_) was measured during the whole experiment in 15 min intervals.

## 3. Results

### 3.1. Analysis of the Amino Acid Sequence of Tgl Protein

**Figure 1:**
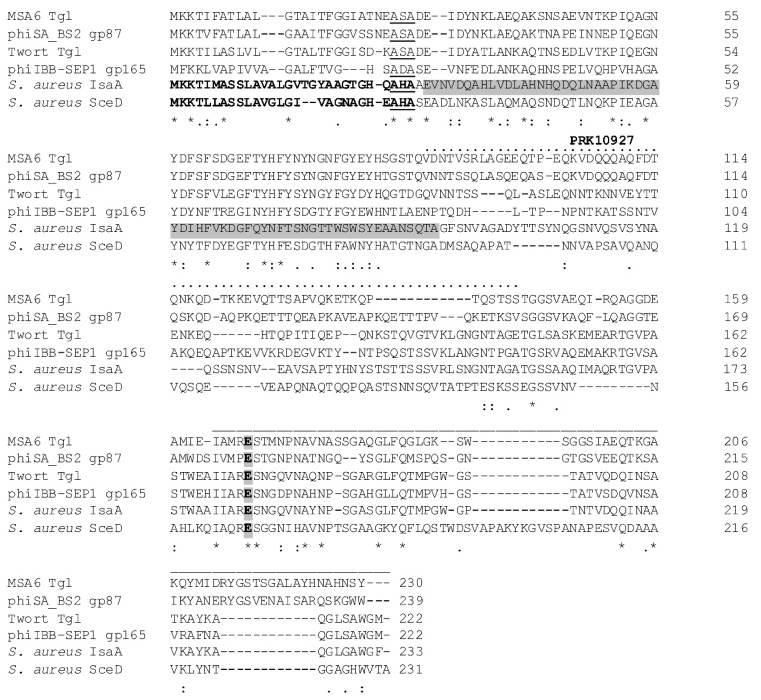
Alignment of amino acid sequences of MSA6 Tgl and its homologs encoded by type phages of *Baoshanvirus*, *Twortvirus*, and *Sepunavirus* genus [5,77,78] with *S. aureus* SceD and IsaA. The Clustal Omega program (CLUSTAL O (1.2.4), https://www.ebi.ac.uk/Tools/msa/clustalo/) [79] served to make the alignment. Protein designations are the following: Tgl- AFN38714, phage phiSA_BS2 phiSABS2_87- AVR55531, phage phiIBB-SEP1 gp165- YP_009601090, phage Twort Tgl- YP_238708.1, IsaA- WP_130826635, SceD- WP_000752008. Signal peptides of IsaA and SceD preproteins are in bold, according to [18,69]. AXA sequences of known or predicted recognition sites for type I signal peptidase are underlined. Regions of known or predicted core lysozyme-like domains are indicated by the line above the alignment, and those of predicted PRK motif by the dotted line above the alignment. Amino acid residues that are identical in all proteins are marked with an asterisk, and those of similar properties are marked with double or single dots. The conserved glutamate known as essential for catalytic activity of lytic proteins with core lysozyme-like domains is highlighted in gray. The region of IsaA sequence essential for the binding to staphylococcal cell wall is highlighted in gray.

Analysis of the 230-aa Tgl protein sequence reveals that it contains a core lysozyme-like domain motif at its C-terminal end (cd13925, pos. 165–230, E = 3.76 × 10^−4^) with a conserved glutamate, which is involved in the catalytic activity of core lysozyme-like domain-containing proteins, including *E. coli* soluble lytic transglycosylase Slt (Figure 1) [68]. Additionally, Tgl contains a predicted signal peptide at its N-terminal end, and the central part of the Tgl sequence contains a motif characteristic of essential cell division protein FtsN (PRK10927, pos. 86–143, E = 2.47 × 10^−3^).

Regions of homology between Tgl and staphylococcal autolysins SceD and IsaA span nearly the whole Tgl sequence (Figure 1). The first of them (pos. 1–27) overlaps with the sequences of SceD and IsaA preprotein signal peptides and includes the AXA motif essential for the recognition and cleavage of SceD and IsaA by the by staphylococcal type I signal peptidase SpsB [69,70]. The second region (pos. 28–89) corresponds to the region of IsaA essential for binding to cell wall [35]. The third region homologous to the PRK10927 motif in Tgl is the least conserved. In turn, the motif of core lysozyme-like domain of resuscitation-promoting factor proteins is common for IsaA, SceD, and Tgl. It overlaps with the motif of lysozyme-like domains (cI00222; E = 3.66 × 10^−3^ in Tgl, E = 2.60 × 10^−14^ in SceD, and E = 1.83 × 10^−5^ in IsaA). The latter is common for a large family of enzymes involved in the cleavage of beta-1,4-linked polysaccharides, including SLTs, goose egg-white lysozyme (GEWL), and bacteriophage lambda endolysin. When searched with HHpred against protein structure database, the Tgl region encompassing the motif of lysozyme-like domains appeared to be significantly similar to active enzyme domains of *Pseudomonas aeruginosa* MltF (4P0G; E = 1.1 × 10^−8^; 5A5X_B, E = 4.8 × 10^−8^), *Escherichia coli* MltC (4CFP_B, E = 4.7 × 10^−8^), *Ralstonia* sp. GH23 family chitinase A-471, *Neisseria meningitidis* LtgA (5O29_A, E = 9.1 × 10^−8^), *E. coli* Slt70 (1QSA_A, E = 1.1 × 10^−7^), hen egg-white lysozyme (2VB1_A, E = 5.2 × 10^−8^), *P. aeruginosa* Slt (5OHU_A, E = 3.1 × 10^−7^), *Campylobacter jejuni* Cj0943 (6CF8_A, E = 1.5 × 10^−7^), goose-type lysozyme (4G9S_A, E = 9.2 × 10^−7^), *E. coli* EtgA (4XP8_A, E = 1.1 × 10^−6^), and *E. coli* MltE (6GI4_B, E = 2.1 × 10^−6^). All of the aforementioned enzymes, except lysozymes and *Ralstonia* sp. GH23 family chitinase, which has an atypical structure [71], belong to LTs of Gram-negative bacteria with LT domains classified as family 1 [72,73]. All these LTs, as well as SceD, IsaA, and phage homologs of Tgl, have been classified as LTs of the GH23 family of glycoside hydrolyses according to the Carbohydrate-Active enZYmes (CAZy) database classification (http://www.cazy.org/) [74]. Consistently, although in general the predicted secondary structure and the region of catalytic glutamic acid residue (at pos. 165 of Tgl sequence) aligns well with the corresponding regions of cod, goose, and swan lysozymes, the region containing two aspartic acid residues (at pos. 90 and 101 of cod lysozyme) critical for the catalytic activity of lysozymes and absent in LTs [75,76] is not conserved in Tgl (data not shown), indicating that Tgl is a lytic transglycosylase.

In addition to *S. aureus* IsaA and SceD and their homologs in *S. aureus-*related bacterial species, proteins that are similar to Tgl over its entire length are encoded only by *S. aureus* phages of the *Kayvirus*, *Twortvirus*, *Sepunavirus* [5], and *Baoshanvirus* [77] genus of the *Twortvirinae* subfamily, as verified by Tblastn [58] (Figure 1). Similarities between Tgl and its closest homologs in these phages are: 100/99–100, 89/44, 82–89/31–33, and 82/56, indicated by % coverage/% identity. *Twortvirinae* of *Silviavirus* [5] and *Sciuriunavirus* [78] genus encode proteins of short regions slightly similar to the core lysosyme-like domain-containing region of Tgl (19–30 coverage/43–44 identity), but they do not contain signal sequences, are products of genes encoded by structural genome modules, and are annotated as tail-tip proteins or tail lysins (data not shown).

### 3.2. Localization of Tgl Protein in S. aureus

**Figure 2:**
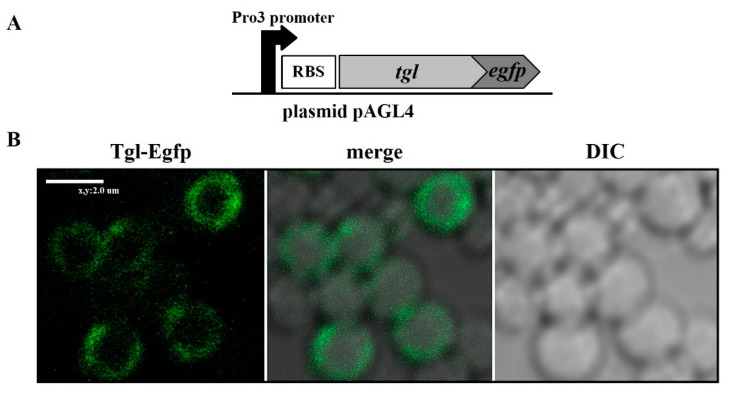
Localization of Tgl–Egfp protein in *S. aureus* cells. (**A**) Schematic picture of *tgl*-*egfp* expression cassette in pAGL4 plasmid. (**B**) Confocal microscope images (left panel) and differential interference contrast (DIC) images (right panel) of *S. aureus* cells producing Tgl-Egfp protein. Central panel shows the merging of both images.

To test whether the homology of Tgl to SceD and IsaA in the region of SceD and IsaA signal peptide sequences reflects the ability of Tgl to be transported to or through the cytoplasmic membrane, we constructed a translational fusion of *tgl* gene to the gene encoding a fluorescent protein, Egfp. In the *S. aureus* cells producing Tgl–Egfp fusion protein from the resultant plasmid (pAGL4), the concentration of fluorescence was detected in cell peripheries, indicating the functionality of the predicted Tgl signal peptide in the transport of Tgl (Figure 2).

### 3.3. Influence of Tgl on the Growth and Survivability of E. coli and S. aureus

**Figure 3:**
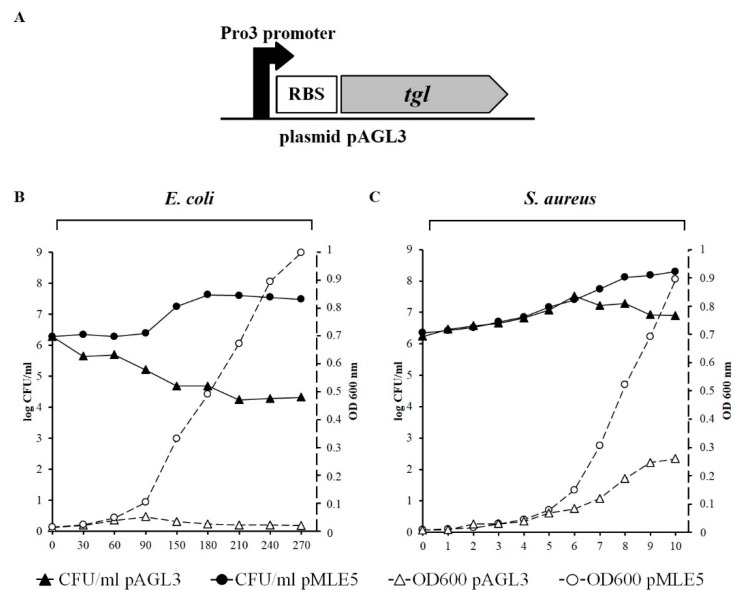
Influence of intracellular Tgl protein on growth and survivability of *E. coli* DH5α and *S. aureus* RN4220 cells. (**A**) Schematic picture of *tgl* expression cassette in pAGL3 plasmid. (**B**,**C**) Changes in optical density (dotted lines) and number of colony forming units (solid lines) in cultures of *E. coli* DH5α (**B**) and *S. aureus* RN4220 (**C**) cells harboring pAGL3 or pMLE5 plasmid (empty control vector). At zero time, the diluted overnight culture of each strain (1:100; OD_600_ of about 0.001) was transferred to 42 °C to derepress transcription from the Pro3 promoter. Left y-axis of each graph represents the log of CFU/mL, right y-axis represents OD_600_. Each curve shows representative results of one of three independent experiments.

Attempts to clone the *tgl* gene in *E. coli* in a shuttle *E. coli*–*S. aureus* vector, pAGL2, under the control of constitutive promoter were unsuccessful. Thus, to analyze the *tgl* function, we cloned *tgl* in a shuttle pMLE5 vector under the control of thermoinducible promoter Pro3 (Figure 3A, Figure S1).

Induction of the *tgl* transcription in *E. coli* cells carrying the resultant plasmid, pAGL3, caused cell lysis, as indicated by a decrease in the optical density of cell culture and a decrease in the number of viable cells (CFU/mL; Figure 3B). Clearly, Tgl can cause lysis of *E. coli* cells from within, indicating that it has muralytic activity and can pass the cytoplasmic membrane to get access to the *E. coli* cell wall. The induction of *tgl* expression from the same plasmid in *S. aureus* cells also caused a decrease in the number of viable cells in culture but to a much lesser extent and with a long delay as compared to the decrease observed in the case of *E. coli* (Figure 3C). Moreover, the optical density of *S. aureus* cells expressing *tgl* grew much slower than that of cells with the empty vector, but no decrease of optical density was observed even in the 10th hour upon the induction of *tgl* expression. The pMLE5 plasmid, whose backbone is derived from the shuttle pSK5630 vector (Figure S1), is driven by a high-copy-number ColE1 plasmid replicon in *E. coli* cells, but by the low-copy-number replicon of pSK1 plasmid in *S. aureus* cells [55]. Despite the copy-number differences, the retention of pSK5630 backbone-based plasmids in *E. coli* and in *S. aureus* is similar (Figure S2). However, we observed that the level of β-galactosidase measured in *S. aureus* and *E. coli* cells harboring the pMLE3 plasmid, parental for pMLE5 and containing the *lacZ* gene under the control of Pro3 promoter, is nearly three times higher in *E. coli* than in *S. aureus* (Figure S3). Thus, the observed differences between the responses of *E. coli* and *S. aureus* to the induction of *tgl* expression may be due, at least in part, to the differences between the levels of *tgl* transcription in *E. coli* and *S. aureus* carrying pAGL3.

### 3.4. Muralytic Activity of Tgl Protein against S. aureus Cell Walls

**Figure 4:**
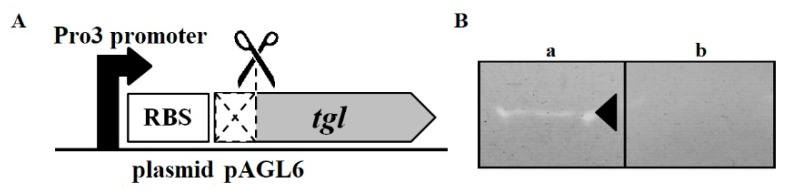
*S. aureus* cell wall-degrading activity of Tgl protein. (**A**) Schematic picture of expression cassette of pAGL6 plasmid encoding recombinant version of Tgl (TglΔSP) protein deprived of signal peptide. (**B**) Zymograms obtained upon separation of pellet proteins of *E. coli* BL21 cells harboring plasmid pAGL6 (a) or pMLE5 (empty control vector) (b), by SDS-PAGE in gel containing dead *S. aureus* cells. Band of clearing that was detected upon protein renaturation in the gel (see Materials and Methods), is indicated by arrowhead.

To overproduce the Tgl protein in *E. coli* cells, we attempted to eliminate the problem of Tgl lethality for *E. coli* by creating a version of Tgl (TglΔSP) depleted of the N-terminal 28 amino acid residues comprising the signal peptide (Figure 4A). *E. coli* cells expressing the recombinant *tgl* gene from the pAGL6 plasmid, in contrast to control cells with an empty vector, produced a protein of predicted molecular mass of TglΔSP (24 kDa), indicating that it was TglΔSP (Figure S4). The majority of the TglΔSP protein was in the cell pellet.

To test whether TglΔSP could act as an *S. aureus* cell wall degrading protein, extracts from crude preparations of induced *E. coli* BL21 strain cells harboring plasmid pAGL6 were subjected to denaturing polyacrylamide gel electrophoresis in gels containing *S. aureus* cell walls. Protein extracts of cells harboring pMLE5 plasmid (empty vector) served as negative control in this experiment. After the renaturation of proteins in the gel and gel staining with Coomassie blue, a clear lysis zone on a blue background was observed in the region of predicted TglΔSP localization, in lanes containing pellet proteins of the lysed *E. coli* BL21/pAGL6 cells. No similar zone was present in lanes with control (Figure 4B and Figure S5). Clearly, the Tgl protein can act as an *S. aureus* cell wall hydrolase. Additionally, our results indicate that the N-terminal 28-aa region of Tgl is dispensable for the ability of Tgl to degrade the *S. aureus* cell wall.

### 3.5. Influence of Nisin on Growth and Survivability of Tgl Producing S. aureus Cells

**Figure 5:**
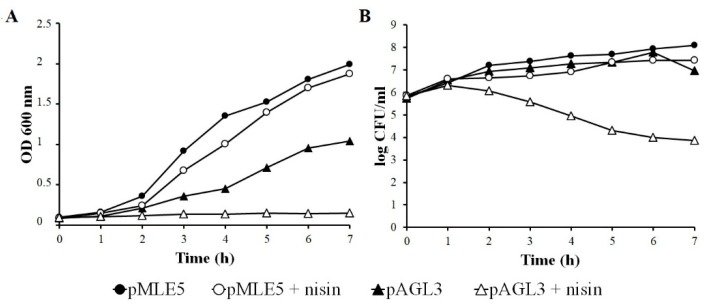
Effect of intracellular Tgl protein on (**A**) growth and (**B**) survivability of nisin-treated *S. aureus* cells. Overnight cultures of *S. aureus* cells bearing pAGL3 (*tgl*+) or pMLE5 plasmid (empty control vector) were diluted 1:100 in LB and incubated at 30 °C until reaching optical density (OD_600_) of about 0.1. At time zero, cultures were transferred to 42 °C, supplemented with nisin concentrate (50 µL per 25 mL *S. aureus* culture), and incubated further. Parallel samples without nisin served as controls. Each curve shows representative result of one of three independent experiments.

The hydrolytic activity of TglΔSP against *S. aureus* cell wall suggests that the intact Tgl protein can function as an additional endolysin of *Twortvirinae* genera phages that encode it. To verify whether Tgl can lyse *S. aureus* cells from within, we supplemented a culture of *S. aureus* producing Tgl with nisin. Nisin is a membrane-permeabilizing bacteriocin produced by certain *Lactococcus* strains. It forms pores in the bacterial cell membrane and dissipates the proton motif force (pmf) [80], thus mimicking the action of phage holins. While nisin at the concentration used did not inhibit the growth and did not decrease the survivability of *S. aureus* cells with pMLE5 plasmid (empty vector), in the case of cells carrying the TglΔSP-encoding plasmid (pAGL3) it inhibited the increase of the culture density and caused a decrease in the number of viable cells in the culture (Figure 5). Taken together, our results indicate that Tgl can function as an endolysin in *S. aureus* cells, but requires the help of pore-forming protein to get access to *S. aureus* cell wall. In cells infected with a Tgl-encoding *Kayvirus* genus phage, this help is likely to be provided by a phage-encoded holin.

### 3.6. Analysis of Transcriptional Activity of Putative Early tgl Promoter

In the genomes of *Twortvirinae* that encode Tgl protein, the *tgl* gene is preceded by a sequence that is similar to promoters for housekeeping RNA polymerase of *S. aureus* cells (Figure S6) [6,81].

**Figure 6:**
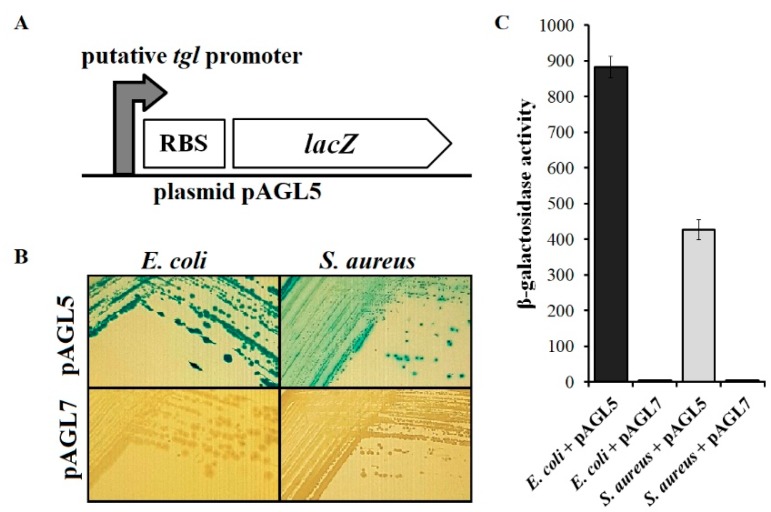
Transcriptional activity in *E. coli* and *S. aureus* of the region preceding the MSA6 *tgl* gene. (**A**) Schematic picture of transcriptional fusion in pAGL5 plasmid. Coordinates in the genome of MSA6 of promoter region cloned are 32,540–33,289 bp. (**B**) Colonies formed on LB solid medium with x-gal at 30 °C by *E. coli* DH5α and *S. aureus* RN4220 cells that were transformed with pAGL5 or pAGL7 plasmid (control). (**C**) Activity of β-galactosidase in cells of *E. coli* DH5α and *S. aureus* RN4220 bearing pAGL5 or pAGL7 plasmid. Activity of β-galactosidase in *S. aureus* cells with plasmids was normalized by the subtraction of background that has been observed in *S. aureus* RN4220 and results from yellow coloration of 2-aminophenoxazin-3-one, produced by the majority of *S. aureus* strains [82]. Each value is the average of results of at least three independent assays; vertical bars show average deviations.

To verify whether this sequence can function as an early promoter of *tgl*, we inserted it upstream of the promoterless reporter gene, *lacZ,* in the pAGL7 plasmid (Figure 6A). *E. coli* as well as *S. aureus* cells with the resultant plasmid (pAGL5) formed blue colonies on the solid LB medium supplemented with x-gal, indicating that the inserted DNA fragment can drive the transcription of reporter gene in both of these bacterial species in the absence of any phage proteins (Figure 6B). Consistently, the activity of β-galactosidase in cells of *E. coli* and *S. aureus* containing the pAGL5 plasmid reached a few hundred Miller units, while in cells with the empty vector no β-galactosidase activity was detected (Figure 6C). Clearly, the *tgl* gene can be transcribed from an early phage promoter, which is dependent on the host RNA polymerase with a housekeeping sigma factor. Although the activity of β-galactosidase in *E. coli* cells with pAGL5 appeared to be twice as high as in *S. aureus* cells with this plasmid, the difference between the copy numbers of pAGL5 in *E. coli* as compared to *S. aureus* cells is likely to be a contributing factor.

### 3.7. Susceptibility of Tgl-Producing S. aureus to Vancomycin

**Figure 7:**
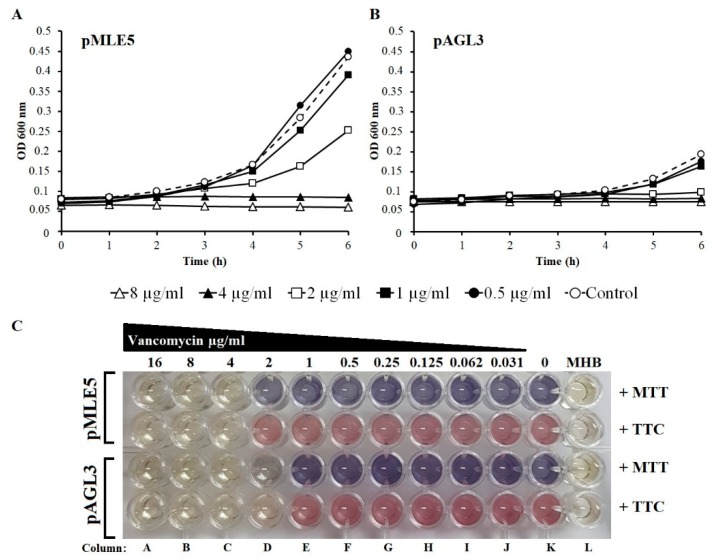
Influence of intracellular Tgl protein on susceptibility of *S. aureus* to vancomycin. (**A**) Growth of Tgl producing *S. aureus* RN4220 cells in the presence of vancomycin at various concentrations. Overnight cultures of *S. aureus* cells bearing pAGL3 (*tgl*^+^) or pMLE5 plasmid (empty control vector) were diluted 1:100 in Muller Hinton broth (MHB) supplemented with chloramphenicol (20 µg/mL) and incubated with shaking for 1 h at 30 °C and an additional 1 h at 42 °C (to derepress the expression of cloned *tgl* gene). At time zero, 100 μL portions of cultures were transferred to wells of pre-warmed (42 °C) honeycomb plates containing 100 μL MHB supplemented with vancomycin at concentrations indicated. Plates were incubated in a Bioscreen C Microbiology Plate Reader for 6 h at 42 °C with medium-intensity shaking. Optical density of samples (OD_600_) was measured during the whole experiment in 15 min intervals. Each curve shows representative result of one of three independent experiments. (**B**) Determination of minimum inhibitory concentrations (MICs) of vancomycin for *S. aureus* cells producing Tgl protein. MICs of vancomycin were determined in MHB using two-fold dilution plate method, as described previously [67], with some modifications. Briefly, 100 μL portions of RN4220 cell cultures containing pAGL3 or pMLE5 (as indicated) and prepared as in (**C**) were transferred to wells (columns A–K) of prewarmed (42 °C) honeycomb plates containing 100 μL MHB with (column A–J) or without (column K) vancomycin. Column L contained 200 µL of MHB without antibiotic as control for medium sterility. Plates were incubated for 5 h at 42 °C. After incubation, 66 µL of dimethyl thiazolyl diphenyltetrazolium bromide (MTT) (0.3 mg/mL) or triphenyl tetrazolium chloride (TTC) (0.1%) per well were added (as indicated) and plates were incubated for 90 min at 37 °C with gentle shaking (110 rpm). MICs were read manually. Comparison between columns L and K shows a change of natural color of MTT and TTC diluted in MHB (respectively yellow and colorless, column L) to the reduced form (respectively violet and red, column K).

The increased production of *S. aureus* SceD protein was shown to correlate with hVISA or VISA phenotype [48,83,84] which in turn is associated with vancomycin treatment failure [85,86]. Thus, Cui et al. [17] speculated that the expression of staphylococcal phage homologs of SceD during *S. aureus* infection may increase the minimum inhibitory concentration (MIC) of vancomycin required to treat infection by MRSA. To verify this possibility, we compared the MICs of vancomycin required to inhibit the growth of *S. aureus* RN4220 expressing the *tgl* gene from a plasmid with that of RN4220 carrying an empty vector. We did not observe any decrease in the susceptibility of *S. aureus* producing Tgl to vancomycin compared to the control strain (Figure 7). On the contrary, we reproducibly observed a slightly increased susceptibility to vancomycin of *S. aureus* producing Tgl compared to that with an empty vector (MIC change from 4 to 2), indicating that Tgl can decrease the *S. aureus* tolerance to vancomycin.

## 4. Discussion

Phage therapy is a promising alternative to antibiotic therapy to cure infections caused by drug-resistant *S. aureus* strains [87]. However, each potential therapeutic phage should be well characterized at the structural and functional level before introducing it for wider use, to ensure the safety of therapy [16,17]. Although representatives of *Twortvirinae* subfamily phages that proved to be effective in curing infections with *S. aureus* have been characterized at the level of genomic sequence, the function of over half of their genes is unknown [6,17,88,89]. Of special interest is the *tgl* gene, whose predicted product is slightly homologous to major surface antigens and virulence factors of *S. aureus* cells, SceD and IsaA [6]. Both of them act as autolysins and have been classified as lytic transglycosylases based on the presence of a motif characteristic of lytic transcglycosylase domain (pfam06737) in their C-terminal parts [18]. Here we show that homologies of Tgl with SceD and IsaA, which span the region of lytic transglycosylase motif at protein C-terminal domains and overlap the region of pre-SceD and pre-IsaA signal peptide sequences, correlate with functional similarities. Like SceD and IsaA [18], Tgl protein can digest peptidoglycan of *S. aureus* cell wall. Also like SceD and IsaA [18,90], Tgl can be transported through the cytoplasmic membrane by a bacterial transport system, as indicated by its localization in the *S. aureus* cell peripheries and its ability to cause lysis of *E. coli* cells from within. High homologies of the predicted Tgl signal peptide to the signal peptides of SceD and IsaA, including the conserved AXA motif essential for the cleavage by type I signal peptidases (SPases), suggests that Tgl can be also translocated through the cytoplasmic membrane by these SPases with the removal of signal peptide. Consistently, we show that the N-terminal fragment of Tgl, corresponding to the predicted signal peptide, is dispensable for the muralytic activity of Tgl.

The physiological significance of PRK10927 motif, characteristic for FtsN proteins, in the amino acid sequences of Tgl is unclear. *S. aureus* does not encode any obvious homolog of FtsN or proteins that directly interact with FtsN in Gram-negative bacteria. Additionally, the amino acid residues that are essential for the function of FtsN are absent from Tgl, SceD, and IsaA (data not shown). Possibly, this region can facilitate the interaction of Tgl with cell wall, as has been proposed for the corresponding region of IsaA [35].

**Figure 8:**
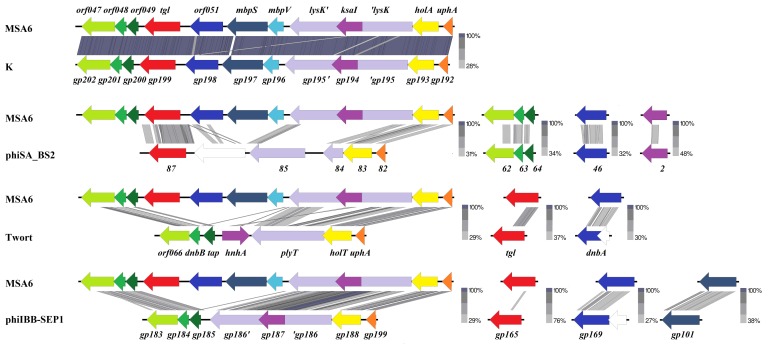
Similarities and differences in the organization of genome regions containing lytic modules and *tgl* gene homologs in the genomes of kayviruses (MSA6 and K, GB acc no. JX080304 and KF766114, respectively) and representatives of *Baoshanvirus* (phiSA_BS2, GB acc no. MH028956) *Twortvirus* (Twort, GB acc. no. MT151386), and *Sepunavirus* genus (phiIBB_SEP1, GB acc. no. NC_041928) of *Twortvirinae.* Genes encoding homologous proteins are marked by similar colors.

In the genomes of staphylococcal kayviruses, the *tgl* gene is located in the cluster of leftward transcribed genes three genes downstream of *lysK*, which encodes the major endolysin of these phages (Figure S6 and Figure 8) [89]. LysK protein contains two domains associated with its muralytic activity: amidase-2 family domain (N-acetylmuramoyl-l-alanine amidase) and CHAP domain (cysteine, histidine-dependent amidohydrolase/peptidase) [91]. They cleave the *S. aureus* peptidoglycan at the amide bond between the *N*-acetylmuramic acid (MurNAc) of glycan moiety and the l-alanine of peptide side chain, and at the peptide bond between D-alanine and the first glycine of the pentaglycine cross-bridge, respectively [92]. As a canonical endolysin, which relays on the function of pore-forming holin to get access to the cell wall, LysK does not contain any signal peptide or transmembrane domain, and its gene is immediately preceded by a holin-encoding gene (*holA*). Based on the close neighborhood of *tgl* gene to *lysK* and *hol*, and on the confirmed mureinolytic activity of Tgl protein, we postulate that *tgl* is an additional component of the kayvirus lytic module and encodes a second endolysin of these phages. The conserved structural and amino acid sequence motifs of family 1 lytic transglycosylases in the Tgl protein indicate that Tgl cleaves the glycan component of cell wall peptidoglycan, and that the cleavage occurs on the reducing side of *N*-acetylmuramic acid. In this respect Tgl resembles the lambda phage endolysin (product of gene R) and the *Pseudomonas* phiKZ phage endolysin Gp144, which are also lytic transglycosylases (see [72,93,94] and references therein).

Several phage endolysins containing two domains of muralytic activity have been identified experimentally or by bioinformatics analysis of predicted products of phage genes (reviewed by [61,95] and references therein). However, fewer examples of phages are known that encode two endolysins targeting different bonds in peptidoglycan. In general, combinations of lysins of different cleavage specificity and lysins of multiple muralytic domains of different cleavage specificity perform better in the disruption of bacterial cell walls than enzymes of single muralytic domain when acting alone (reviewed by [95]). Consistently, a cocktail of LysK and lysostaphin and also engineered LysK enriched with the glycyl-glycine M23 endopeptidase domain of lysostaphin outperformed either of these combination components in the ability to lyse *S. aureus* [96,97]. Two endolysins of different cleavage specificity may also extend the phage host range, as was shown for *Bacillus thuringiensis* phage GIL01 [98]. The production by kayviruses of two endolysins, LysK and Tgl, targeting three kinds of bonds in peptidoglycan may be a feature of these phages contributing to their high lytic efficacy and wide host range.

Proteins of phage lytic modules fulfill their function at the last step of phage development. However, typically, the expression of endolysin genes and, in certain cases, the secretion of endolysins to the cell wall environment start at an early stage of infection and reach maximum levels at late stages (see [99] and references therein, [100]). The *tgl* gene is preceded by a sequence that is similar to promoters for host RNA polymerase with a housekeeping sigma factor [6,82]. Indeed, we show here that it is transcribed by a host RNA polymerase in the absence of any phage proteins, a hallmark of early phage genes. Meanwhile, the transcripts of *lysK* were detected in phage-infected cells between 10 and 20 min after phage infection, as shown in studies with type kayvirus K (unpublished data referred to in [91]). Consistently, neither *lysK* nor *holA* genes are preceded by any sequence resembling promoters for bacterial RNA polymerase holoenzyme [6]. We conclude that the transcription of *tgl* gene is at least in part independent of the transcription of *lysK* and starts early in phage development.

Despite the early production of Tgl protein and its likely host SpsB-dependent transport through the cytoplasmic membrane, kayvirus-infected cells do not lyse prematurely. This is consistent with the proposed dependence of endolysin-mediated cell lysis on prior dissipation of membrane proton motif force (pmf) and cell death, which is normally triggered by holins and was shown to be a holin function additional to the formation of membrane pores [101]. According to this lysis model, even an endolysin accumulated in the extracytoplasmic environment acts ineffectively until holin-mediated dissipation of the pmf at a defined time can cause cell death and prevent the repair of endolysin-mediated lesions by cellular proteins. In support of that, the genomes of phages predicted to encode only secretory endolysins harbor a holin gene [100,102]. Our results fully conform to this model. Although *S. aureus* cells producing Tgl from the cloned *tgl* gene stopped growing, they did not lyse unless their culture was supplemented with nisin, a peptide antibiotic that forms pores in the cytoplasmic membrane and dissipates the pmf, thus mimicking the function of holins. Similar phenomena were observed in the case of the other secreted endolysins: Lys44 of *Oenococcus oeni* bacteriophage fOg44, which contains SP, and LysPP1 of *Bacillus subtilis* phage SPP1, which was artificially enriched with SP [100,101,102]. Exponentially growing cells of Gram-positive hosts (*O. oenni* or *L. lactis,* and *B. subtilis,* respectively) producing either of these endolysins from a cloned gene or treated with externally added endolysin at physiological concentrations lysed only if their culture was supplemented with nisin or exposed to other factors or conditions dissipating the membrane pmf.

The dependence of intracellular Tgl on nisin in the lysis of *S. aureus* cells that was observed in this work contrasts with the ability of Tgl to cause rapid lysis of *E. coli* cells in the absence of nisin. This difference is likely to result from a lower level of *tgl* gene expression in *S. aureus* as compared to *E. coli* in our experimental system and from the requirement of more enzyme for the disruption of *S. aureus* peptidoglycan, which is much thicker than that of *E. coli*. In the case of SPP1 bacteriophage endolysin enriched with SP, the requirement of nisin for cell lysis also depended on the concentration of endolysin [103]. While at physiological endolysin concentration lysis required nisin, it could occur in the absence of nisin when the endolysin concentration was several times higher.

The *tgl*, *lysK*, and *holA* genes are in the region encompassing eight genes between the functionally different genome modules, of which one encodes tRNAs and the other ribonuclease and structural virion components (Figure 8) [6]. Of the three genes between *lysK* and *tgl,* two, *mbpV* and *mbpS*, encode predicted membrane proteins. MbpV is a 102-aa protein with a transmembrane domain in its N-terminal part and numerous positively charged amino acid residues in its C-terminal moiety (data not shown). In this respect it resembles holins. The amino acid sequence of MbpS contains a conserved SPFH domain motif (PF01145) characteristic for stomatin, prohibitin, flotillin, and HflK/C proteins [104]. Additionally, the predicted secondary structure of MbpS in the region between amino acid residues 57 and 244 is highly similar to that of archeal stomatin of *Pyrococcus horikoshi* (3BK6_C; 4 × 10^−19^) as indicated by results of HHpred analysis. In bacterial cells, stomatins, flotillins, and related proteins are parts of the so-called functional membrane microdomains (FMMs), which are similar to the lipid rafts of eukaryotic cells [105,106,107]. FMMs allow for compartmentalization of different processes within the membrane despite their close proximity [106]. The main component of lipid rafts is flotillin, a scaffold protein that recruits other proteins and stabilizes their multimeric complexes [105]. One of the most represented FMM-associated proteins is SecA, as was shown for *S. aureus*, *B. subtilis*, and *Borrelia burgdorferi* [106]. The interaction of Sec secretion machinery with flotillins is important for Sec-associated protease and protein secretion activities, as was shown in the case of *B. subtilis* [106,108]. One cannot exclude that in *Staphylococcus* cells infected with a *Kayvirus*, MbpS protein facilitates the SpsB(Sec)-mediated transport of Tgl through the membrane.

The organization of the *tgl* and *lysK* gene region appears to be similar to that in MSA6 in all *Kayvirus* genus phages genomes (Figure 8). In the genomes of *Twortvirus*, *Baoshanvirus*, and *Sepunavirus* genus phages, which also encode a homolog of Tgl, this organization is slightly different, indicating genomic rearrangements. Genomes of *Silviavirus* and *Sciuriunavirus* genus do not encode a homolog of Tgl. Conceivably the *tgl* gene was acquired by phages of certain *Twortvirinae* genera from their bacterial host after their separation from a common ancestor, and evolved to serve bactericidal functions. Production of two endolysins targeting different bonds in peptidoglycan may be advantageous for *Twortvirinae*. Lytic modules of *Silviavirus and Sciuriunavirus* genus phages, which do not encode a homolog of Tgl protein, also encode two proteins of predicted muralytic activity [88,109,110,111].

The homology of Tgl protein to *S. aureus* autolysins SceD and IsaA has been a major concern. Cui et al. [17] wondered whether *S. aureus* cells infected with a *Kayvirus* genus phage and producing Tgl could be less sensitive to vancomycin than non-infected cells. While this danger is irrelevant to normal kayvirus development, which leads to cell lysis, it may be relevant to cases of temporal maintenance of lytic phage genome at a stage of multiplication inhibition in slowly growing or starved bacterial cells, commonly known as pseudolysogeny and associated with the expression of certain phage genes and the loss of phage DNA by some pseudolysogens (see [112,113] and references therein). Our preliminary results suggest that kayviruses can form a pseudolysogenic relationship with their bacterial host. However, we do not confirm the possibility of decreased sensitivity to vancomycin by Tgl-producing *S. aureus*, which might apply to pseudolysogens. On the contrary, we demonstrate here that *S. aureus* cells producing Tgl are less tolerant to vancomycin than their Tgl-free counterparts. The amino acid sequence of Tgl is only 36% identical to that of SceD over 82% of the entire protein length. Conceivably, the influence of Tgl on *S. aureus* cells is limited to mureinolysis.

Certain natural or engineered phage endolysins of amidase or endopeptidases activity were shown to act synergistically with vancomycin to kill *S. aureus* cells [114,115,116,117]. The influence of endolysins of lytic transglycosylase activity on the sensitivity to vancomycin has not been studied previously. Our results show for the first time that endolysins of this activity can also act as allies in the fight with pathogens of reduced vancomycin sensitivity.

## 5. Conclusions

Enzymes of phage and bacterial origin, responsible for the cleavage of the same kinds of bonds in peptidoglycan, have enzymatic domains of similar sequence or structure, indicating the past transfer of their ancestral genes between bacteria and phages. However, the further fates of these genes in evolution are tightly associated with the needs of the genomes that carry them. Staphylococcal phages of certain *Twortvirinae* genera, kayviruses among them, encode a distant homolog (Tgl) of two secreted autolysins and virulence factors of *S. aureus* (IsaA and SceD)*,* which they apparently acquired after the differentiation of *Twortvirinae*. Here we show that, like IsaA and SceD, Tgl has features of lytic transglycosylase and can disrupt *S. aureus* and *E. coli* cell walls. Like IsaA and SceD, it is secreted through the cytoplasmic membrane with the help of its signal peptide and host signal peptidases of type I. However, the location of *tgl* gene in a genome lytic module of phages that have this gene indicates that Tgl protein functions as a phage endolysin. Like other secreted endolysins, Tgl, when not overproduced, requires a dissipation of pmf to cause cell lysis. Early expression of *tgl* gene in phage development and lytic transglycosylase properties of Tgl protein imply that Tgl can support the function of the major kayviral endolysin LysK, which is a late phage protein and has amidase and cysteine, histidine-dependent amidohydrolase/peptidase activity. Possibly, the combined ability of Tgl and LysK to target three kinds of bonds in cell wall peptidoglycan contributes to the wide host range and high lytic efficacy of kayviruses and related *Twortvirinae*. Most importantly, we show here that the production of Tgl by *S. aureus*, unlike the production of SceD, is not associated with decreased sensitivity to vancomycin but causes decreased tolerance to vancomycin.

## Supplementary Materials

The following are available online at https://www.mdpi.com/1999-4915/12/3/292/s1. Supplementary experimental procedure: Plasmid retention test. Table S1: Oligonucleotides used in this study. Figure S1: Organization of genes and regulatory sites in the *S. aureus*–*E. coli* shuttle expression vector pMLE2 and its derivatives. Figure S2: Retention of pSK5630, pDAS122, pMLE3, or pAGL1 plasmid in *E. coli* DH5α and *S. aureus* RN4220 cells grown in the absence of selection for the resident plasmids. Figure S3: Activity of β-galactosidase in *E. coli* DH5α and *S. aureus* RN4220 cells containing pDAS122, pMLE3, or pAGL1 plasmid. Figure S4: Overproduction of recombinant version of Tgl protein (TglΔSP) deprived of N-terminal fragment corresponding to the signal peptide. Figure S5: *S. aureus* cell wall destruction activity of TglΔSP protein (full image). Figure S6: Genetic organization of *tgl* gene region of *Kayvirus* genome (based on Łobocka [6]).
