# Supplementary material for: A Kayvirus Distant Homolog of Staphylococcal Virulence Determinants and VISA Biomarker Is a Phage Lytic Enzyme
Source: Viruses. 2020 Mar 7;12(3):292. doi: 10.3390/v12030292 (PMC7150955; doi:10.3390/v12030292)
Supplement: Supplementary file 1 [file viruses-12-00292-s001.pdf]

viruses

MDPI

Supplementary Material

A *Kayvirus* distant homolog of staphylococcal virulence determinats and VISA biomarker is a phage lytic enzyme

Aleksandra Głowacka-Rutkowska[1], Magdalena Ulatowska[1], Joanna Empel[2], Magdalena Kowalczyk[1], Jakub Boreczek[1], Małgorzata Łobocka[1*]

[1] Institute of Biochemistry and Biophysics of the Polish Academy of Sciences, Warsaw, Poland; glowacka@ibb.waw.pl (A.G.-R.); miulatowska@gmail.com (M.U.); mk@ibb.waw.pl (M.K.); jakub.boreczek@ibb.waw.pl (J.B.); lobocka@ibb.waw.pl (M. Ł.)

[2] Department of Epidemiology and Clinical Microbiology, National Medicines Institute, Warsaw, Poland; j.empel@nil.gov.pl (J.E.)

* Correspondence: lobocka@ibb.waw.pl; Tel.: +48-22-592-1300

1. Supplementary experimental procedures

1.1. Plasmid retention test

Plasmid retention was assayed as described previously, with some modifications [1]. *E. coli* DH5α or *S. aureus* RN4220 cells, bearing the pMLE5, pDAS122, pSK5630 or pAGL1 plasmid were grown overnight in LB medium supplemented with ampicillin (100 µg/mL, *E. coli*) or chloramphenicol (20 µg/mL, *S. aureus*) at 37 °C. Overnight cultures were diluted 1:100 in similar medium and incubated at 37 °C with shaking (200 rpm) until the optical density (OD_{600}) of about 0.3. Next, the cultures were serially diluted in LB, spread onto LB agar plates without antibiotic, and incubated overnight at 37 °C. Cells of single colonies were suspended in 1 ml portions of LB, serially diluted in LB and spread onto LB agar plates without antibiotic. Plates were incubated for 24 h at 37 ºC, and colonies grown were replica plated onto a solid LB medium with an antibiotic selective for a given plasmid and without antibiotic. Plasmid retention was expressed in % as the fraction of colonies able to grow on antibiotic-containing medium. Calculations of plasmid retention were based on colony counts for that fraction of the population that represented the progeny of carriers of the tested plasmid, as verified by testing the antibiotic resistance of cells in undiluted suspensions.

2. Supplementary Tables

2.1 Oligonucleotides used in this study

**Name**	**Oligonucleotide sequence 5’ → 3’**
OMLS001F	CGCACTAGTAAAAAGCTGTTGACATATTGCTCTAATAATGTTATTATTAT AGCAATCAC GTGTAAGGAG
OMLS002R	CGCTCATCGATAATTTCACCG
pMLE1F	CTAGTAAAAAGCTGTTGACATATTGCTCTAATAATGTTATTATAGCAATC ACGTGTAA GGAGGTGAAAAATCGATGCATG
pMLE2R	CATCGATTTTTCACCTCCTTACACGTGATTGCTATAATAACATTATTAGAG CAATATGTCAACAGCTTTTTA
pMLE3F	CGATTCGAGCTCGGTGCACCGGTTCTA GAGCATG
pMLE4R	CTCTAGAACCGGTGCACCGAGCTCGAAT
OAGL39	TATAGCATGCTTAATAACTATTATGTGCGTTGT
OAGL40	TATAATCGATATGAAGAAAACAATTTTTGCAAC
OAGL49	TATAACCGGTATAACTATTATGTGCGTTGT
OAGL57	TATACACGTGAAATTTAAAACTCCTTAAATAATTTTTTAGAATTGTTTATT TG
OAGL78	TATAGGCGCCATGTATGAATGTTTATGTGAATGCGGAGAAG
OAGL108	TATAATCGATATGGAAATTGATTATAATAAGTTAGCAGA
OAGL109	TATAGCATGCTTAGTGGTGGTGGTGGTGGTGGCCGCCGCCATAACTATTA TGTGCGTTGTGGTATG

3. Supplementary Figures

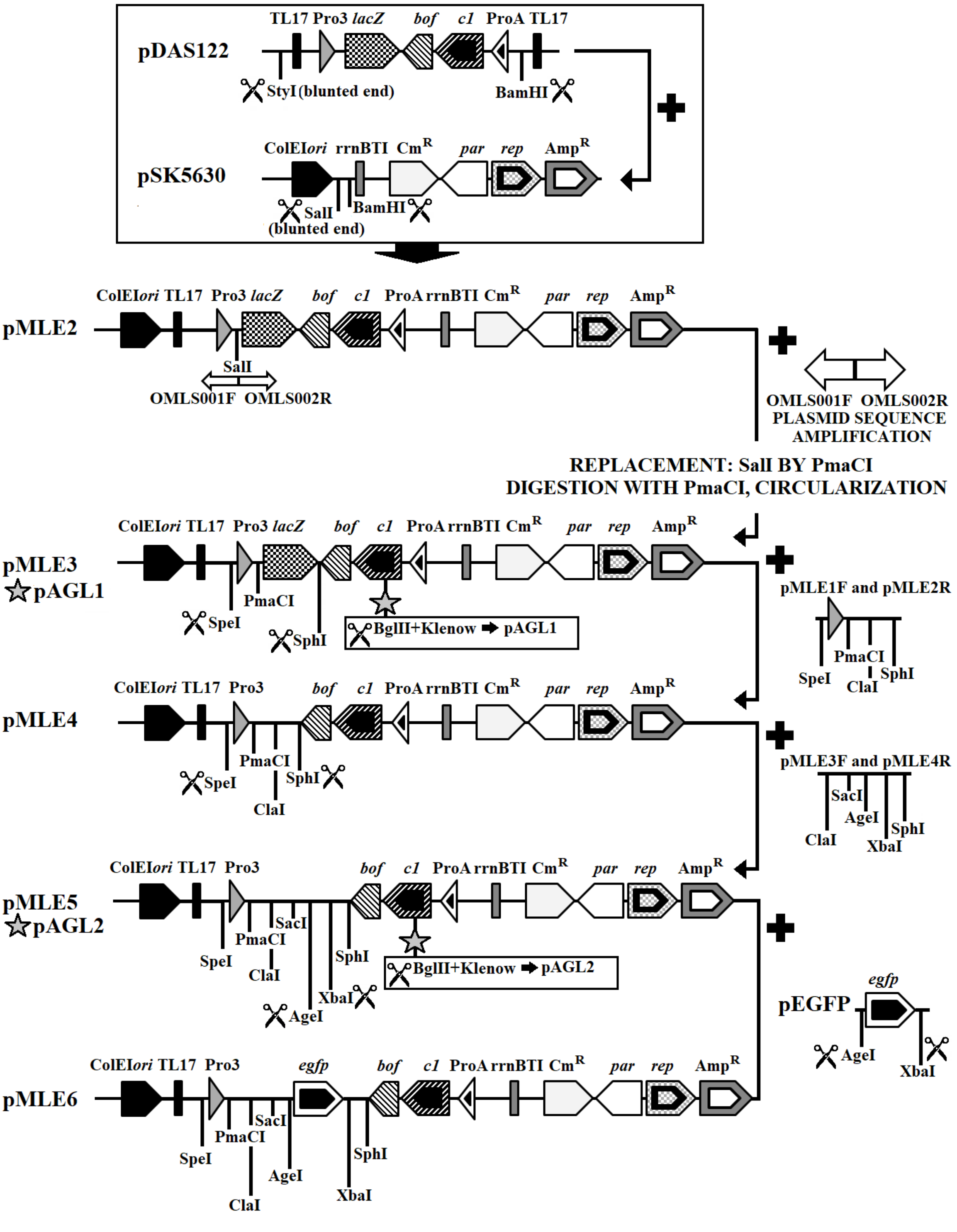

Supplementary Figure S1. Organization of genes and regulatory sites in the *S. aureus*/ *E. coli* shuttle expression vector pMLE2 and its derivatives. The starting shuttle *E. coli* – *S. aureus* plasmid pMLE2 was constructed by ligation of the core sequences of

shuttle vector pSK5630 [2] with the expression cassette of pDAS122 plasmid [3] (see Materials and Methods). The expression cassette is separated from the remaining parts of the vector by two sets of tandem transcription terminators, which are indicated as black and grey boxes. The cloning site for genes of interest is preceded by the synthetic promoter (Pro3). Transcription from Pro3 is regulated by the temperature-sensitive C1 repressor of phage P1 and by P1 Bof (Lxc) protein, which modulates the C1-mediated repression. The expression of *c1* and *bof* genes in the vector is under the control of constitutive promoter ProA. Constitutive expression vectors pAGL1 and pAGL2 were constructed by the introduction of frameshift mutation into the *c1* gene P1 in the pMLE3 or pMLE5 plasmid, respectively (the respective pAGL1/pAGL2 mutation is indicated by a grey star).

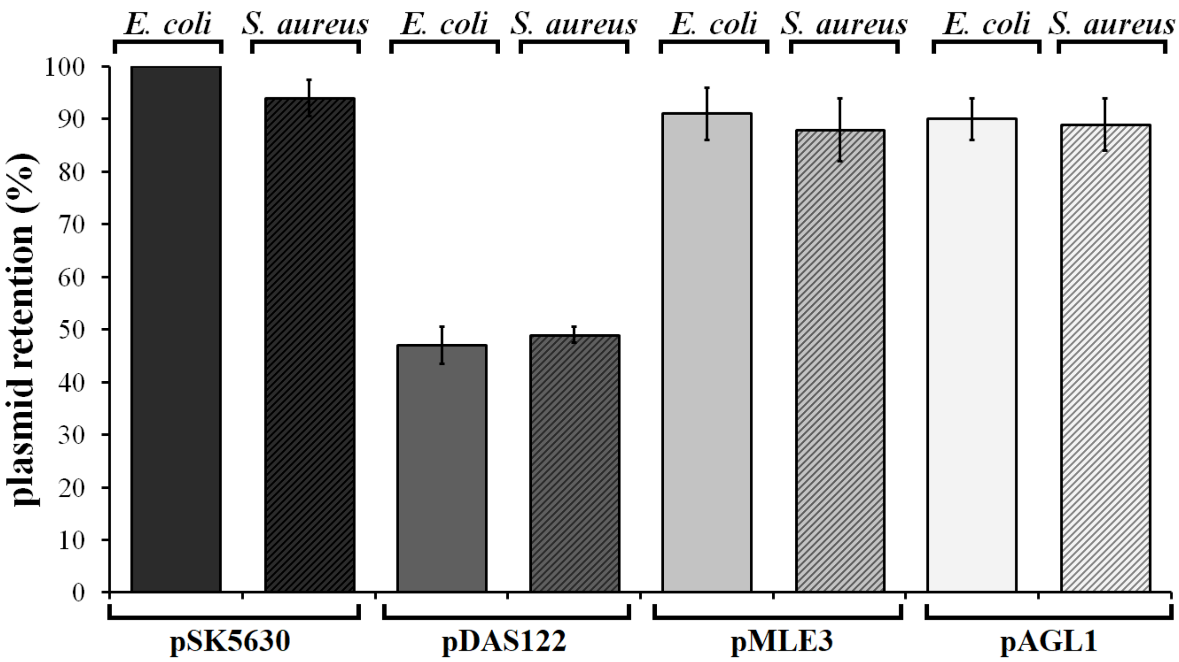

Supplementary Figure S2. Retention of the pSK5630, pDAS122 , pMLE3 or pAGL1 plasmid in *E. coli* DH5α and *S. aureus* RN4220 strain cells grown in the absence of selection for the resident plasmids. The retention is expressed in % as the ratio of the number of plasmid-containing cells to all cells in a colony grown for about 23 generations in the absence of selection for the resident plasmid. Each value is the average of results of at least three independent assays; vertical bars show average deviations.

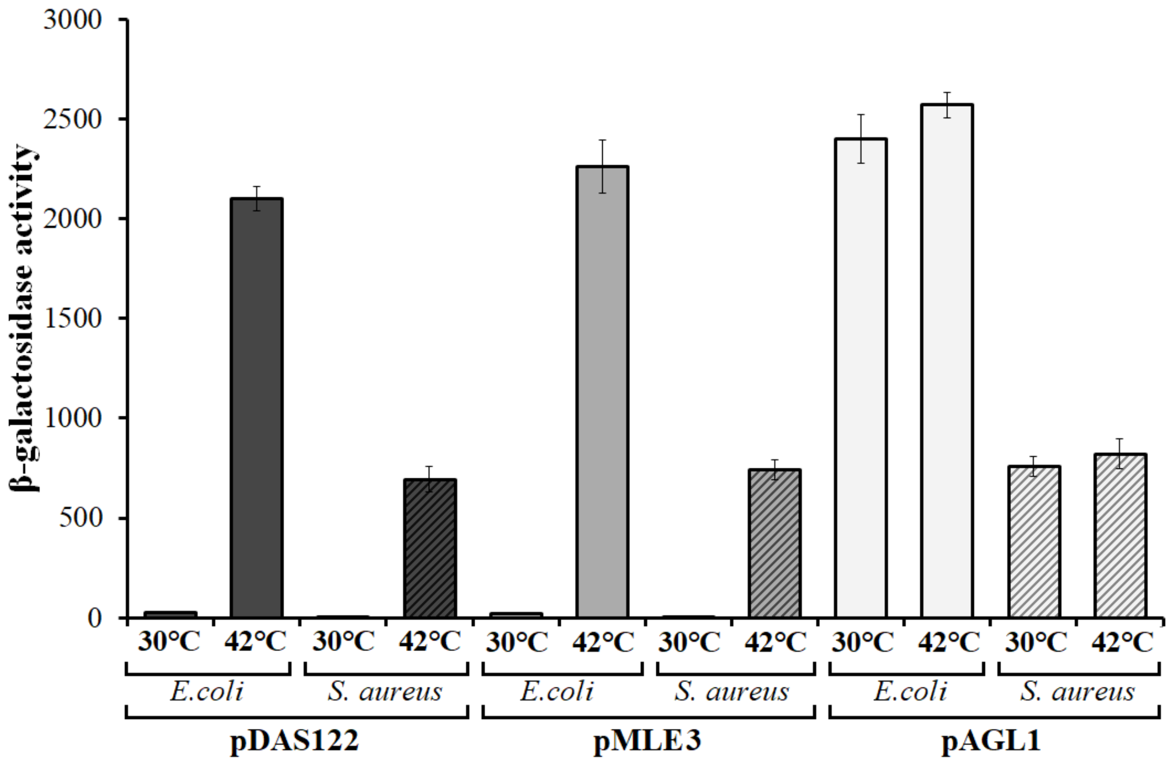

Supplementary Figure S3. Activity of β-galactosidase in *E. coli* DH5α and *S. aureus* RN4220 strain cells containing pDAS122, pMLE3 or pAGL1 plasmid. Cell cultures for the assay were incubated at 30 °C or 42 °C (as indicated) until the optical density (OD_{600}) of about 0.3. Activities of β-galactosidase were determined by Miller's method. The activity of β-galactosidase in *S. aureus* cells with plasmids was normalized by the subtraction of background that has been observed in *S. aureus* RN4220 and results from yellow colouration of 2-aminophenoxazin-3-one that is produced by the majority of *S. aureus* strains [4]. Each value is the average of results of at least three independent assays; vertical bars show average deviations from the mean.

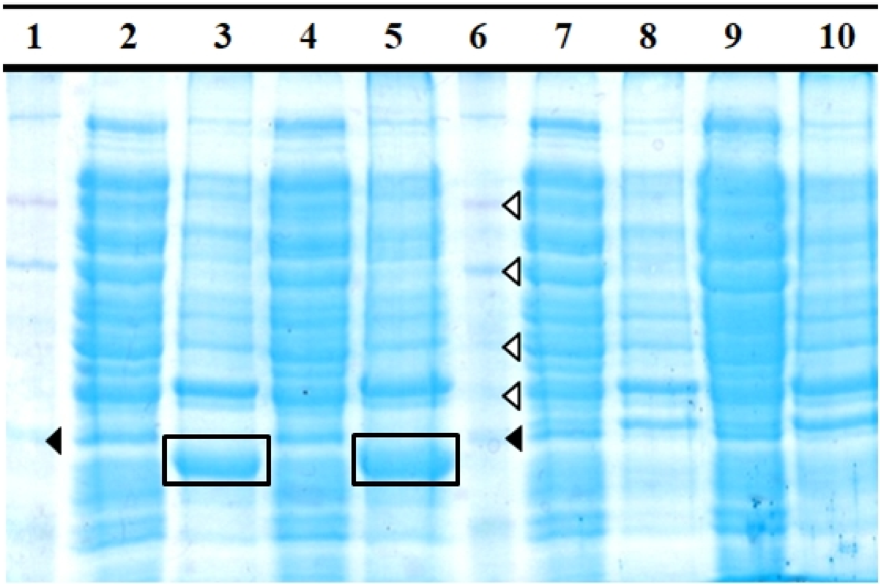

Supplementary Figure S4. Overproduction of recombinant version of Tgl (TglΔSP) protein deprived of N-terminal fragment corresponding to the signal peptide. Designations of lanes in a gel are the following: 1 and 6 - size marker proteins (ColorPlus Prestained Protein Marker, Broad Range 7-175 kDa, NewEngland BioLabs, Cat. No. P7709); 2 to 5 - proteins of *E. coli* BL21 cells bearing the pAGL6 plasmid; 7 to 10 - proteins of cells bearing the pMLE5 plasmid (an empty control vector). Lanes 2; 4; 7 and 9 - represent proteins of the supernatant obtained after bacterial cells lysis and centrifugation. Lanes 3; 5; 8 and 10 - represent proteins of the pellet obtained after bacterial cells lysis and centrifugation, dissolved in buffer with 8 M urea. The bands representing TglΔSP protein are boxed. White arrowheads indicate (in order from the top) 80, 58, 46, 30 kDa marker fragments. Black arrowheads indicate 23 kDa marker fragment.

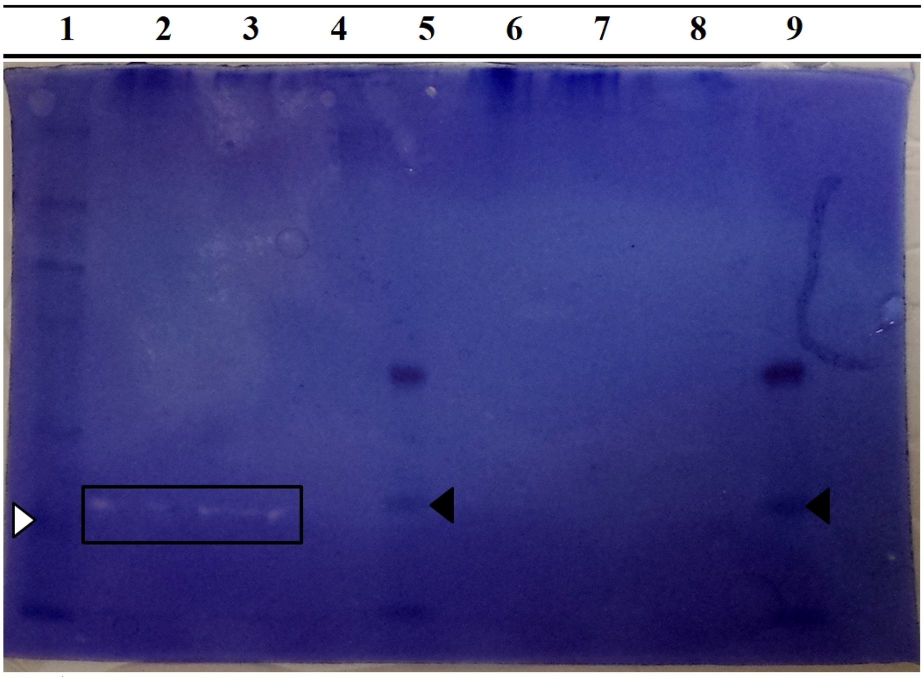

Supplementary Figure S5. *S. aureus* cell wall distruction activity of the TglΔSP protein (full image). Designations of lanes in a gel are the following: 1 - size marker proteins (ColorPlus Prestained Protein Marker, Broad Range 7-175 kDa, NewEngland BioLabs, Cat. No. P7709); 5 and 9 - size marker proteins (Spectra Multicolor Low Range Protein Ladder, Thermo Scientific, Cat. No. 26628); 2 and 3 - proteins of the pellet obtained after lysis and centrifugation of *E. coli* BL21 cells bearing the pAGL6 (*tglΔSP*$^{+}$) plasmid, dissolved in buffer with 8 M urea; 4 and 8 - empty lanes; 6 and 7 - proteins of cells bearing pMLE5 plasmid (an empty control vector) obtained as above. Lysis zones were observed only in protein extracts from crude preparations of cells producing the recombinant version of the Tgl (TglΔSP) protein. The localization of bands representing proteins with lytic activity (boxed) is in agreement with the predicted molecular weigth of TglΔSP protein (23.45kDa). White and black arrowheads indicate 23 and 25 kDa marker fragments, respectively.

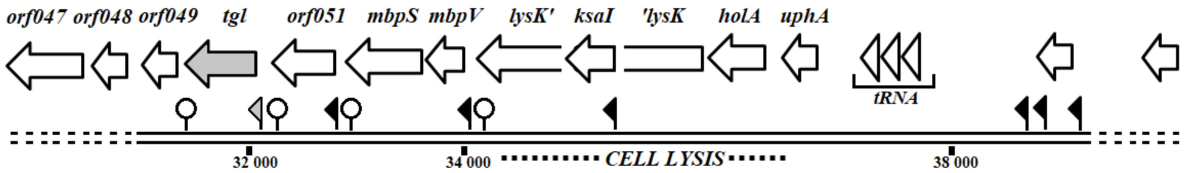

Supplementary Figure S6. Genetic organization of the *tgl* gene region of *Kayvirus* genome [based on 5]**.** Arrows mark the position and orientation of genes. The *tgl* gene (depicted as a grey arrow) is located in the cluster of leftward transcribed genes, three genes downstream of intron-interrupted *lysK*, which encodes endolysin and five genes downstream of *holA*, which encodes a holin. The predicted early *tgl* promoter is represented by a grey flag, while the predicted early promoters of the remaining genes are represented by black flags over the double line, which represents DNA [5,6]. Lolipops indicate predicted Rho-independent transcription terminators.

4. Supplementary References

1. Grigoriev, P.S.; Łobocka, M.B. Determinants of segregational stability of the linear plasmid-prophage N15 of *Escherichia coli*. *Mol Microbiol.* **2001**, *42*, 355-68.

2. Grkovic, S.; Brown, M.H,; Hardie, K.M.; Firth, N.; Skurray, R.A. Stable low-copy-number *Staphylococcus aureus* shuttle vectors. *Microbiology*. **2003**, *149*, 785-94.

3. Schofield, D.A.; Westwater, C.; Hoel, B.D.; Werner, P.A.; Norris, J.S.; Schmidt, M.G. Development of a thermally regulated broad-spectrum promoter system for use in pathogenic gram-positive species. *Appl Environ Microbiol.* **2003**, *69*, 3385-92.

4. Tse, H.; Chan, E.; Lam, C.W.; Leung, K.F.; Chow, P.; Lee, K.C.; Sze, K.H.; Cheung, S.K.; Tse, M.K.; Ho, P.L.; Leung, S.P.; Lau, S.K.; Woo, P.C.; Yuen, K.Y. Production

of 2-aminophenoxazin-3-one by *Staphylococcus aureus* causes false-positive results in β-galactosidase assays. *J Clin Microbiol.* **2012**, *50*, 3780-2.

5. Łobocka, M.B.; Hejnowicz, M.S.; Dąbrowski, K.; Gozdek, A.; Kosakowski, J.; Witkowska, M.; Ulatowska, M.; Weber-Dąbrowska, B.; Kwiatek, M.; Parasion, S.; Gawor, J.; Kosowska, H.; Głowacka, A. Genomics of staphylococcal Twort-like phages: potential therapeutics of the post-antibiotic era. *Adv Virus Res.* **2012**, *83*, 143-216.

6. Vandersteegen, K.; Kropinski, A.M.; Nash, J.H.; Noben, J.P.; Hermans, K.; Lavigne, R. Romulus and Remus, two phage isolates representing a distinct clade within the Twortlikevirus genus, display suitable properties for phage therapy applications. *J Virol.* **2013**, *87*, 3237-47.
